# A well-rested scalpel: a proposal for standardized guidelines on surgeon fatigue

**DOI:** 10.3389/frhs.2025.1713346

**Published:** 2025-11-20

**Authors:** Dat Tien Le, Mohammad Najm Dadam, Ethar Shaaban, Dang Xuan Thang, Mukhammadbektosh Khaydarov, Phillip Tran, Nguyen Tien Huy, Tran Cong Duy Long

**Affiliations:** 1 Department of Hepatobiliary and Pancreatic Surgery, University Medical Center at Ho Chi Minh City, Ho Chi Minh City, Vietnam; 2 Department of Surgery, Faculty of Medicine, University of Medicine and Pharmacy at Ho Chi Minh City, Ho Chi Minh City, Vietnam; 3 Helios Klinikum Schwelm, Department of Orthopedics and Trauma Surgery, Schwelm, Germany; 4 Online Research Club, Nagasaki, Japan; 5 Faculty of Medicine Delta University for Science and Technology, Gamasa, Egypt; 6 Cardiology Department, Ha Nam General Hospital, Ninh Binh, Vietnam; 7 Institute of Research and Development, Duy Tan University, Da Nang, Vietnam; 8 School of Medicine and Pharmacy, Duy Tan University, Da Nang, Vietnam; 9 Department of Surgery, AKFA Medline University Hospital, Tashkent, Uzbekistan; 10 School of Medicine, Central Asian University, Tashkent, Uzbekistan; 11 Invasive Cardiology Nam Can Tho University, Can Tho, Vietnam; 12 School of Tropical Medicine and Global Health (TMGH), Nagasaki University, Sakamoto, Nagasaki, Japan

**Keywords:** surgeon fatigue, burnout, patients safety, fatigue countermeasures, perioperative safety, surgical performance

## Abstract

A significant gap in perioperative safety persists due to the absence of internationally recognized guidelines for managing surgeon fatigue. While other high-risk fields utilize robust fatigue management systems, surgical institutions frequently rely on fragmented coping strategies and inconsistent local policies. This oversight is concerning, as evidence confirms that sleep deprivation compromises surgical performance, with simulator studies reporting technical skill reductions of up to 32%. Current countermeasures, such as work-hour limits or caffeine use, are insufficient substitutes for restorative sleep and have an inconclusive impact on patient care. This paper proposes a systemic solution, urging global, national, and hospital-level collaboration to establish a standardized framework for fatigue risk management. Key recommendations include the use of fatigue-monitoring tools, mandating rest periods that allow for at least six hours of sleep before elective procedures, creating backup on-call rosters, and making fatigue management a part of surgical training. Adopting these evidence-based protocols is an essential step toward protecting patients and fostering a sustainable, safer surgical culture.

## Graphical Abstract

**Figure:**
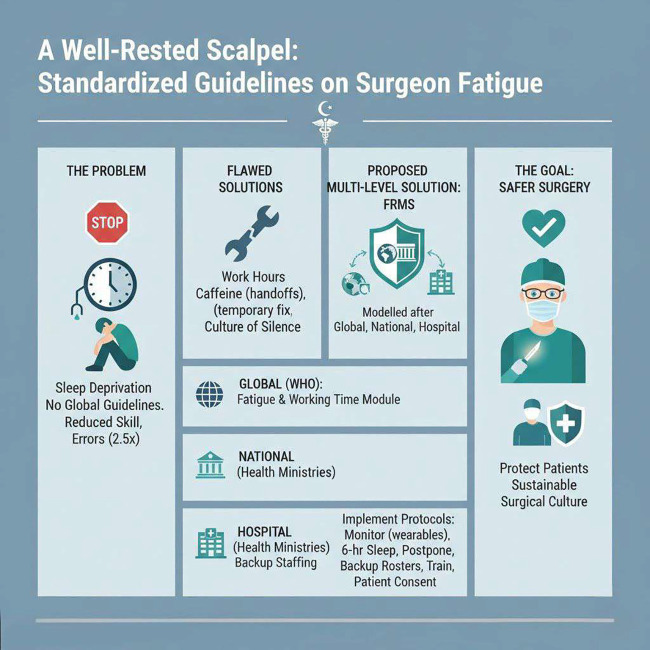

## 1 Introduction

Sleep deprivation among surgeons represents a critical and preventable threat to patient safety and physician well-being. Surgical practice is inherently demanding, involving long hours, night shifts, unpredictable emergencies, and high cognitive load, which often results in inadequate rest. Evidence from both military and medical domains demonstrates that within 48 h of sustained wakefulness, individuals may become functionally “combat-ineffective” (1). Fatigue reduces attention, memory, and decision-making, while also impairing dexterity and prolonging operative times (2, 3).

Despite decades of evidence on the risks of surgeon fatigue, there remains no internationally recognized guideline to systematically address sleep deprivation in surgery. Instead, institutions continue to rely on fragmented, *ad hoc* coping strategies and inconsistent local policies.

This lack of consensus suggests an important gap in perioperative safety, which can lead to bad outcomes. We urge the World Health Organization (WHO), national health ministries, and hospitals to collaborate to develop standardized fatigue management guidelines to protect both patients and surgical teams.

## 2 The impact of sleep deprivation on surgical performance

Multiple lines of evidence underscore that sleep deprivation compromises surgical performance. Simulator studies report reductions of 11%–32% in technical performance, with more errors, longer operative times, and diminished motion economy (4). Sleep loss further impairs decision-making, multitasking (5), and higher-order functions such as attention, while more basic functions like simple working memory and cognitive flexibility may remain intact (6). Real-world outcomes remain mixed: about one-third of clinical studies report increased complication rates among fatigued surgeons, while others find no significant differences in mortality or complication rates (3). However, large-scale data indicate that surgeons experiencing high burnout had a 2.5-fold greater odds of being involved in a medical error, while emotional exhaustion alone increased this risk by 1.7-fold (7). These inconsistencies may partly reflect methodological challenges, such as reliance on crude outcome measures that overlook subtle cognitive deficits or behavioral deficits. Experience matters, as junior surgeons and residents are more susceptible to fatigue-related impairments (8). Senior surgeons may partially compensate through experience and structured preparation (9), though often at the cost of their long-term well-being (10). The overall consensus remains clear: fatigue impairs performance in ways that are ethically and professionally indefensible if left unaddressed (11).

## 3 Current coping mechanisms and their limitations

There are many techniques to combat fatigue used by surgeons, and these techniques are mostly incomplete. The limitations on work hours set by the ACGME and the EWTD reduce fatigue; however, their impact on patient care is inconclusive. Patient care may actually be worsened by these policies because handoffs are more frequent and less continuous (12). The above methods, as well as individual strategies such as coffee, bright light, and microbreaks, are improvements. However, these strategies are not replacements for sleep (13). Caffeine may improve wakefulness but also can lead to performance detriment in highly skilled tasks at higher doses (14). Sleep banking is a method where there is sleep extension prior to a period of anticipated deprivation. This method is used in the military and in aviation, but in healthcare, its potential is vastly ignored (15). The aim of this paper is to argue that incorporating structured sleep banking schedules into surgical routines will assist in alleviating fatigue and its associated risks. Pharmacologic stimulants, such as modafinil and amphetamines, can increase alertness; however, the user may become overconfident and believe there is no room for further lethargy (16). There is a lack of cultural awareness regarding the surgical field. This culture, which encourages lack of endurance and silence surrounding fatigue, socially constructs fatigue in a very dangerous manner (17). The gaps in this approach, which is remaining underdeveloped at the moment, are very important.

## 4 The case for standardized guidelines

In line with practices from aviation and other high-risk industries, fatigue management in surgery should encompass several key components (18). These include the establishment of Fatigue Risk Management Systems (FRMS), that integrate structured rostering rules—such as limiting consecutive night shifts, ensuring ≥11 h of rest between duties, and avoiding “quick returns”—as successfully applied by Transport Canada and the FAA/NASA Fatigue Countermeasures Program (19, 20). Surgeons’ sleep deficits should be assessed and monitored using validated self-assessment tools and/or worn monitoring devices (21, 22). Evidence shows that surgeons obtaining fewer than six hours of sleep before elective procedures are 2.7 times more likely to experience intraoperative complications, whereas those who are sleep-deprived despite achieving at least six hours of rest still face a 170% increased risk of adverse events (23, 24). Accordingly, elective procedures should be postponed when fatigue thresholds are exceeded, and hospitals should maintain on-call rosters of qualified surgeons to fill in for overworked and exhausted colleagues. Additional countermeasures proven effective in other high-risk sectors include controlled 20-minute rest breaks (25), moderate caffeine doses (approximately 32–300 mg) administered early in a shift may enhance alertness and vigilance (26), and fatigue-awareness training such as the UK Association of Anaesthetists’ Fight Fatigue campaign and NHS Fatigue Pack (27). Lastly, in the graduate training, fatigue management and recognition should be imbibed into the curricula, and patients should be informed if their operating surgeon is significantly sleep-deprived, supporting informed consent and, when appropriate, the option to reschedule the procedure.

### 4.1 Implementation considerations

When talking about fatigue management from an organizational perspective, it is crucial to look at fatigue management within a global, national, and hospital context simultaneously and in a coordinated manner. The World Health Organization needs to take the Global Patient Safety Action Plan 2021–2030 and augment it with a fatigue and working time module, developing a first model incorporating FRMS principles (28) successfully used in aviation and now adapted to healthcare. At the national level, Ministries of Health must customize and implement these frameworks to the local context, mandating adherence, providing backup staffing, on-call system resources, and alignment with the WHO/ILO occupational health and safety standards (29). At the hospital level, institutions ought to incorporate Fatigue Risk Management Systems, monitor the impact of schedule reforms, and promote the rest of the surgical culture as protective to patients (30). Furthermore, the fundamental barriers, like culture, pushback from senior surgical staff, and lack of health workforce and resources in low- and middle-income and developing countries, make the execution of such plans difficult. High-volume centers can be the starting point and support pilot programs to test the refinement of resistance evidence, sustainability, and feasibility to promote the surge for evidence-based practice.

## 5 Conclusion

For sure, sleep deprivation on the part of the surgeon is an epic risk when it comes to the safety of patients and the doctor himself/herself, both of whom are put in danger and are in dire need of safety and protection, all of which are not in any way an illusion. While the results, when analyzed in the context of certain outcome studies, might not be as straightforward, any simulator-based studies and cognitive or clinical trials show consistent results affirming that performance is impaired in one way or another. Current and existing coping mechanisms for this problem, such as the use of caffeine, short naps, and all peer casual and informal support, are not and have never been effective. It is only the clear and present danger that is resulting from sleep deprivation on the part of the surgeon that is leading to the emergence of global guidelines. Healthcare systems have the power, thanks to the existing fatigue assessment methods, mandatory rest and lap periods, active avoidance of procedures, replacement staffing systems, educational initiatives, and openness, that can lead to safer surgical procedures along with longer sustainable working periods in the field.

## Data Availability

The original contributions presented in the study are included in the article/Supplementary Material, further inquiries can be directed to the corresponding authors.
